# Dentist's Gold

**Published:** 1896-01

**Authors:** 


DENTISTS' GOLD.—The gold which is taken from teeth which have been filled or which is filled or scraped from the gold used in making tooth crowns or new gold fillings is no inconsiderable item for a dentist to consider. A dentist recently said that the sweepings from his carpet had netted him $35 in a single month. The little scraps of gold that remain after many operations are gathered in bottles and sold at a fair price to the dealers from whom the gold is obtained. In some cases the gold is mixed with some other metal, or with dust, but all of it has a commerical value. The price paid by dealers is, of course, much less than that asked for the sheets of gold bought by the dentists.

Gold used in dental operations is of several degrees of fineness. Fourteen-karat gold has its uses in the making of tooth plates, and other finer gold to the value of several hundred dollars at one time is not unusual with a busy member of the profession.—*N. Y. Tribune.*

